# Metabolomics studies of cell–cell interactions using single cell mass spectrometry combined with fluorescence microscopy†

**DOI:** 10.1039/d2sc02298b

**Published:** 2022-05-16

**Authors:** Xingxiu Chen, Zongkai Peng, Zhibo Yang

**Affiliations:** Chemistry and Biochemistry Department, University of Oklahoma Norman Oklahoma 73072 USA zhibo.Yang@ou.edu

## Abstract

Cell–cell interactions are critical for transmitting signals among cells and maintaining their normal functions from the single-cell level to tissues. In cancer studies, interactions between drug-resistant and drug-sensitive cells play an important role in the development of chemotherapy resistance of tumors. As metabolites directly reflect the cell status, metabolomics studies provide insight into cell–cell communication. Mass spectrometry (MS) is a powerful tool for metabolomics studies, and single cell MS (SCMS) analysis can provide unique information for understanding interactions among heterogeneous cells. In the current study, we utilized a direct co-culture system (with cell–cell contact) to study metabolomics of single cells affected by cell–cell interactions in their living status. A fluorescence microscope was utilized to distinguish these two types of cells for SCMS metabolomics studies using the Single-probe SCMS technique under ambient conditions. Our results show that through interactions with drug-resistant cells, drug-sensitive cancer cells acquired significantly increased drug resistance and exhibited drastically altered metabolites. Further investigation found that the increased drug resistance was associated with multiple metabolism regulations in drug-sensitive cells through co-culture such as the upregulation of sphingomyelins lipids and lactic acid and the downregulation of TCA cycle intermediates. The method allows for direct MS metabolomics studies of individual cells labeled with fluorescent proteins or dyes among heterogeneous populations.

## Abstract 2

We combined single cell mass spectrometry and fluorescence microscopy techniques to study metabolites affected by interactions between different types of cells under ambient conditions.

## Graphical abstract

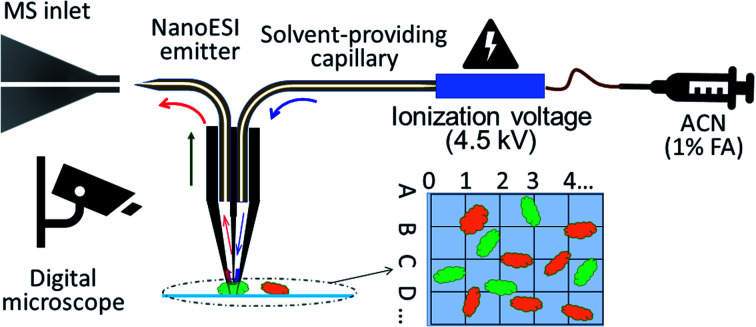

## Introduction

Biological variability of cells not only exists among different types of cells characterized by the expression of specific biomarkers, but is also present in individuals of the same types of cells, where the gene expression, protein synthesis, and cell metabolism are diverse at the single-cell level.^1–4^ In addition, the degree of cell heterogeneity is related to other types of cells in a cellular microenvironment through cell–cell interactions, because the function, survival, and proliferation of cells can be determined by their neighbours in the same niche.^5,6^

The importance of cell–cell heterogeneity has been appreciated in many disease studies, especially drug resistance research in cancer. Resistance to chemotherapy medicine is a major cause of clinical cancer treatment failure.^7^ Understanding drug resistance mechanisms lays the foundation for the development of effective clinical intervention for drug resistance. Over the past few decades, numerous studies have been conducted to tackle molecular mechanisms of resistance in drug-resistant cells. The proposed mechanisms include drug inactivation, drug target alternation, drug efflux, DNA damage repair, epithelial–mesenchymal transition (EMT), and cell death inhibition.^8^ Although drug-resistant cells themselves exhibit resistance to anticancer drugs, their communications with drug-sensitive cells in a tumor microenvironment can enhance chemotherapy resistance of the latter.^9,10^ The mechanisms are attributed to intercellular transfer of molecules, including P-glycoproteins,^11^ midkine,^12^ small RNAs,^13^ and metabolites^10^ that can protect drug-sensitive cells from chemotherapy-induced apoptosis.

There are two major mechanisms of transmitting molecules in cell–cell communication: secreting soluble molecules^14^ (including but not limited to chemokines, cytokines, and growth factors) and transferring extracellular vesicles^15^ (*e.g.*, microvesicles, exosomes, and apoptotic bodies). Different *in vitro* co-culture systems have been developed to study cell–cell interactions, and these systems can be generally classified into two categories: indirect co-culture (*i.e.*, co-culture without cell contact) and direct co-culture (*i.e.*, co-culture with cell contact).^16–20^ In indirect co-culture systems, different types of cells are physically segregated (*e.g.*, cells cultured in different devices or different chambers of the same device^21^), whereas cell–cell communication is through the shared culture medium (*e.g.*, *via* the permeable membrane in a culture device or channels in a microfluidic device^22,23^) or conditioned medium harvested from the other types of cells.^24^ This type of co-culture method is commonly used due to its high reproducibility and commercially available cell culture devices. Particularly, different types of cells can be conveniently separated for molecular analysis. However, due to the lack of direct cell–cell contact, these systems cannot vividly mimic the actual physiological environment, considering that intercellular transfer of some proteins and small RNAs is achieved in a contact- or distance-dependent manner.^25,26^ In contrast, in direct co-culture systems, different types of cells are cultured in the same device with cell–cell contact, indicating that they are better models representing natural cell–cell interactions.^27^ Several techniques (*e.g.*, micropatterning,^28^ temporary divider,^29^ and degradable hydrogel^30^) have been previously developed for indirect co-culture. However, these less-than-ideal methods necessitate a temporary seal between different types of cells, and the intercellular response is hindered due to impacted molecular diffusion between cells.^25^ Alternatively, direct co-culture systems, which allow for direct contact among different types of cells, have been achieved in multiple studies. However, *in situ* molecular analysis of different types of cells is challenging due to heterogeneous cell populations.^20^ Single cell analysis is inevitably needed to overcome these challenges.

As the end products of cellular processes, metabolites directly and sensitively reflect the genetic and environmental changes of cells. Mass spectrometry (MS) has become the major analytical platform for metabolomics studies. Traditional MS metabolomics research, *e.g.*, liquid chromatography-mass spectrometry (LC-MS), relies on samples prepared from populations of cells. Apparently, it is intractable to use these traditional methods for *in situ* studies of the cell–cell interactions among coexisting cells in their living status. Although fluorescence-activated cell sorting (FACS) has been commonly used for cell isolation, this method has a significant impact on cellular metabolism, resulting in altered compositions of metabolites.^31,32^

**Fig. 1:**
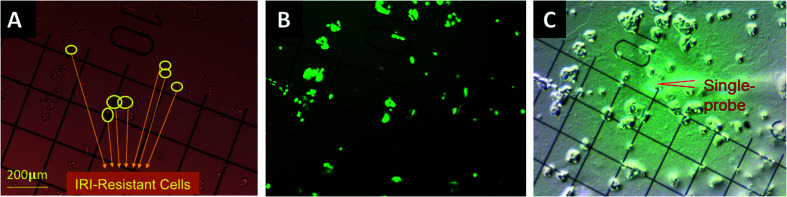
Determination of different types of single cells in the direct co-culture for the Single-probe SCMS analysis. Both drug-sensitive (HCT116-GFP) and drug-resistant (IRI-HCT116) cells were co-cultured and attached on the gridded glass coverslips. Coordinates of single cells in each group were determined by comparing (A) bright-field and (B) fluorescence images of the same coverslip. (C) Metabolomics measurement analysis of both types of cells was conducted using the Single-probe SCMS technique.

The inevitable approach to overcoming the above challenges is to conduct live single cell metabolomics analysis. A variety of sampling and ionization techniques, including vacuum- and ambient-based methods, have been developed for single cell MS (SCMS) metabolomics studies. MALDI (matrix-assisted laser desorption/ionization) and SIMS (secondary ion mass spectrometry), two common vacuum-based ionization techniques, have been used to measure metabolites at the cellular and subcellular scales.^33,34^ Alternatively, the development of ambient-based sampling and ionization techniques (such as live single cell video MS,^35^ probe ESI MS,^36^ nano-DESI MS,^37^ and LAESI MS^38^) created opportunities to study live single cells in their native or near-native environment. We have developed a number of microscale devices, including the Single-probe,^39–45^ T-probe,^46,47^ and micropipette needle,^48^ that can be coupled to a mass spectrometer for SCMS metabolomics studies under ambient conditions. In this work, we designed a new workflow to combine the Single-probe SCMS technique^39^ with fluorescence microscopy to study interactions between drug-sensitive and drug-resistant cells in co-culture systems (Fig. 1). Our experimental setup enables direct *in situ* metabolomics studies of cell–cell interactions, eliminating potential interference of materials (*e.g.*, membranes or dividers needed for cell separation) in cell analysis. Particularly, different types of cells can be labelled with dyes or fluorescent proteins, allowing for selection of the cell of interest in heterogeneous cell populations for SCMS metabolomics studies.

## Experimental methods

### Materials and methods

The device utilized for the indirect co-culture system is a Corning Transwell inserts (Corning Incorporated Life Science, Tewsbury, MA, USA) in the format of 6-well plates with a permeable membrane (pore size: 0.4 μm). The gridded glass coverslips (ibidi USA Incorporation, Fitchburg, WI, USA) were used for cell attachment in the direct co-culture system. Materials to fabricate the Single-probe include a dual-bore quartz tubing (O. D. 500 μm, I. D. 127 μm, Friedrich & Dimmock, Inc. Millville, NJ, USA) and fused silica capillary (O. D. 105 μm, I. D. 40 μm, Polymicro Technologies, Phoenix, AZ, USA). Chemicals used include acetonitrile (EMD Millipore corporation, Burlington, MA, USA), formic acid (Sigma-Aldrich, St. Louis, MO, USA), and irinotecan hydrochloride (BioVision Incorporated, Milpitas, CA, USA). Reagents needed to culture cancer cells include McCoy's 5A cell culture media, penicillin streptomycin (Pen Strep), 0.25% trypsin–EDTA (Life Technologies Corporation, Grand Island, NY, USA), and FBS (Global Life Sciences Solutions, Marlborough, MA, USA).

### Mono-culture systems

Human colorectal cancer HCT116 cells, which are used as the model for drug-sensitive cells in the current study, were purchased from American Type Culture Collection (ATCC; Rockville, MD, USA). HCT116 cells were cultured (at 37 °C in 5% CO_2_) in Petri dishes using McCoy's 5A media supplemented with 10% FBS and 1% Pen Strep. HCT116 cells were split once the confluency reaches 80%.

Multiple methods have been reported to induce drug resistance in cancer cell lines: continuous treatment with stepwise dose, continuous treatment with constant dose, pulsed treatment with stepwise dose, and pulsed treatment with constant dose.^49^ Among them, continuous treatment of chemotherapy is an effective method to induce early-stage resistance. The protocols for preparing the irinotecan (IRI)-resistant cells were adopted from previous publications using continuous drug treatment.^50^ Briefly, HCT116 cells were cultured in a complete growth medium containing 1 μM IRI for 20 days, and cells that survived from this elongated treatment were regarded as IRI-resistant (*i.e.*, IRI-HCT116) cells. To minimize the influence of the intracellular IRI compound on the co-culture systems, the IRI-HCT116 cells were maintained in the regular growth medium for one week to deplete intracellular IRI prior to the co-culture.

Stable HCT116-GFP cells, generated from HCT116 cells by lentiviral vector transduction, were purchased (Cellomics Technology, Halethorpe, MD, USA). Because GFP (green fluorescent protein) labeling has no significant influence on cell metabolism and functions,^51^ HCT116-GFP cells are also defined as drug-sensitive cells in the current study. To maintain the ability of expressing the green fluorescent protein, HCT116-GFP cells were cultured in a complete growth medium containing 1 μg mL^−1^ puromycin. Cells were then maintained in the regular growth medium for three days to deplete intracellular puromycin prior to the co-culture.

### Indirect and direct co-culture systems

Two different types of co-culture systems, *i.e.*, indirect and direct co-culture methods, were utilized to co-culture IRI-resistant (IRI-HCT116) and drug-sensitive (HCT116 or HCT116-GFP) cells.

#### (a) Indirect co-culture

Cells in this co-culture system were cultured using a Corning Transwell inserts combined with 6-well culture plates without direct cell–cell contact. Three different ratios of IRI-HCT116 to HCT116 cells (1 : 1, 5 : 1, and 25 : 1) were used to investigate how the population of drug-resistant cells affect the drug resistance level of drug-sensitive cells. Briefly, IRI-HCT116 cells (1 × 10^5^, 5 × 10^5^, or 2.5 × 10^6^ cells; suspended in 1.5 mL medium) were added into the inserts, whereas HCT116 cells (1 × 10^5^ cells; suspended in 2.6 mL medium) were added in the 6-well plates. The cell-containing inserts and 6-well plates were then combined for co-culture. After being incubated for 3 or 4 days at 37 °C, the HCT116 cells in 6-well plates were harvested for IC_50_ measurement using MTT assay.

#### (b) Direct co-culture

Cells in this co-culture system were cultured with direct cell–cell contact. Both HCT116-GFP and IRI-HCT116 cells were cultured in the same well of a multi-well plate. Two different types of experiments, *i.e.*, fluorescence microscopy observation and SCMS measurement, were performed using these co-cultured cells. First, we monitored cell growth under IRI treatment using a fluorescence microscope. 1400 HCT116-GFP cells (suspended in 40 μl medium) and 1400 IRI-HCT116 cells (suspended in 40 μl medium) were added into the same wells of a 384-well plate. The mixed cells were incubated for one day, treated by IRI (5 or 10 μM), and then observed using a fluorescence microscope to evaluate the density of HCT116-GFP cells. A low magnification objective lens (4×) was used to ensure that all cells in one well of the 384-well plate were captured in a fluorescence image. Images were then processed using ImageJ (ImageJ for windows, Version 1.53m; NIH, Bethasda, MD, USA) to quantify the fluorescence intensities, which were used to compare the relative densities (*i.e.*, viabilities) of HCT116-GFP cells under different treatment conditions. Photos with a higher magnification (with a 10× objective lens) were also captured for better views of single cells. Second, we conducted the SCMS measurements of the direct co-culture cells. 5000 HCT116-GFP and 5000 IRI-HCT116 cells were added into the same wells of a 12-well plate, in which gridded glass coverslips were placed at the bottom. Cells were attached to the glass coverslips after overnight incubation for the Single-probe SCMS analysis.

### MTT assay

The MTT assays were conducted according to the manufacturer's instructions. The HCT116 cells from the indirect co-culture were harvested, rinsed using a fresh culture medium, and seeded into 96-well plates (∼10 000 cells per well). Drug treatments were carried out using different concentrations of IRI (0.1, 1.0, 5.0, 10, or 50 μM). MTT (3-(4,5-dimethylthoazol-2-yl)-2,5-diphenyltetrazolium bromide) was added into each well in 96-well plates, and absorbance values at 570 nm were measured using a microplate reader (Synergy H1, BioTek, Winooski, VT). The IC_50_ values were determined using a Prism (GraphPad Software, San Diego, CA), and Microsoft excel was used to construct dose–response curves.

### LC-MS/MS identification

#### Cell lysate preparation and lipid extraction

The Folch method was adopted to extract lipids from HCT116-GFP cell lines for the LC-MS study. After detaching cells from the Petri dish using trypsin, cells were collected for lipid extraction. 10 000 cells were mixed with 3 mL chloroform/method (2 : 1, v/v), vortexed on ice for 10 min, and then centrifuged for 10 min. The organic layer was collected, dried using a SpeedVac concentrator (SPD111V, Thermo Scientific, San Jose, CA), and then reconstituted in 150 μL chloroform for the following LC-MS analysis.

#### LC-MS

LC-MS/MS was carried out as a complementary method to identify ions of interest. An UltiMate 3000 HPLC system (Thermo Scientific, San Jose, CA) was coupled to an LTQ Orbitrap XL mass spectrometer. A Luca 3u C18 column (50 × 2.00 mm, 3 μm, Phenomenex, Torrance, CA) was used for chromatographic separation. The HPLC conditions were set as follows: injection volume: 5 μl; column oven: 50 °C; flow rate: 350 μl min^−1^; mobile phase A: water/methanol (95/5, v/v); mobile phase B: isopropanol/methanol/water (60/35/5, v/v). Both mobile phases contain 10 mM ammonium formate and 0.1% formic acid. The total run time was 80 min, including 5 min equilibrium. Tandem MS (MS/MS) was carried out in targeted analysis mode, and the normalized collision energy (NCE) was set as 24–25 (manufactory's unit).

### The Single-probe SCMS setup

The Single-probe was fabricated following our published protocols.^39^ Briefly, three major components (*i.e.*, a nano-ESI emitter, a dual-bore quartz tip, and a fused silica capillary) were integrated to prepare a Single-probe. Dual-bore quartz needles were produced by pulling the dual-bore quartz tubing using a laser micropipette puller (Sutter P-2000, Sutter Instrument, Novato, CA). The nano-ESI emitters were pulled from the fused silica capillaries using a butane micro torch. A Single-probe was fabricated by embedding a fused silica capillary and a nano-ESI emitter into these two channels of a dual-bore quartz needle. The Single-probe was then coupled to a Thermo LTQ Orbitrap XL mass spectrometer for SCMS analysis (Fig. 1). The sampling solvent (acetonitrile supplemented with 1% formic acid) was used for the SCMS experiment at a flowrate of ∼0.05 μl min^−1^. The MS parameter settings included mass range *m*/*z* 200–1500 for positive ion mode and *m*/*z* 50–900 for negative ion mode, mass resolution 60 000, ionization voltage 4.5 kV, 1 microscan, and 100 ms max injection time.

### SCMS analyses of cells in direct co-culture

The gridded glass coverslips containing co-cultured IRI-HCT116 and HCT116-GFP cells were used for the SCMS experiments. First, we obtained the optical images of the co-cultured cells to determine the locations of each type of cells on the gridded glass slides. A Nikon Eclipse Ti microscope was used to take both bright-field (Fig. 1A) and fluorescence images (Fig. 1B) of cells attached on the gridded glass coverslips, which are kept in 12-well plates during image capture. Based on the comparison of these two types of images, we manually determined the coordinates of each type of cell on gridded glass cover slips (Fig. 1). Second, the gridded glass coverslips containing cells were taken out from 12-well plates, rinsed with a FBS-free culture medium, and placed onto the XYZ-stage for the Single-probe SCMS measurements (Fig. 1C). Guided by the digital microscope images, we analyzed both types of cells on the gridded glass slides. To determine metabolite change by cell–cell communication in the direct co-culture, the SCMS experiments were also carried out using the mono-cultured IRI-HCT116 and HCT116-GFP cells.

### SCMS data analysis

We adopted our previously established SCMS data analysis workflow, including data pretreatment, statistical analysis, and database searching, to analyze the experimental results.^52^ First, we conducted SCMS data pretreatment, including noise removal, background subtraction, ion intensity normalization, and alignment. Noise removal is performed to remove instrument noise; background subtraction can eliminate contaminants (*e.g.*, ions from the solvent and cell culture medium); ion intensity normalization provides an effective approach to comparing the relative abundances of species from different cells. Although quantitative Single-probe SCMS experiments can be performed to measure the absolute quantity or concentration of target molecules (*e.g.*, anticancer drugs) in single cells, the internal standard (*e.g.*, isotopically labelled anticancer drug) is needed in each study.^41,53^ It is impractical to obtain the absolute quantitative information of large numbers of species from single cells. In our studies, ion intensities were normalized to the total ion current (TIC), a commonly used normalization method in MS studies, and then multiplied by an arbitrary scaling factor of 10^5^. Peak alignment was then performed to correct minor mass shift in different measurements. The first three steps were performed using our customized R script,^52^ whereas peak alignment was achieved using Geena 2.^54^ Second, statistical analyses, including multivariate analysis (*e.g.*, principle component analysis (PCA) and partial least squares-discriminant analysis (PLS-DA)) and univariate analysis (*e.g.*, Student's *t*-test), were conducted using MetaboAnalyst.^55^ Lastly, three online metabolomics databases, Metlin,^56^ HMDB,^57^ and GNPS^58^ were utilized to tentatively label all ions. MS/MS structure verification of ions of interest was conducted through three different approaches: at the single-cell level using the Single-probe SCMS method, MS analysis of cell lysate using direct injection, and LC-MS/MS analysis of cell lysate.

## Results and discussion

### Changes of drug-resistance levels of IRI-resistant cells in the drug-free medium

IRI-HCT116 cells (IC_50_ = 16.6 ± 1.4 μM) were produced by culturing the parental HCT116 in a medium containing low-dose of IRI. It was reported that drug-resistance levels of drug-resistant cells can gradually decline when cultured in a drug-free medium over time.^59^ In our studies, we incubated IRI-HCT116 cells using the culture medium (IRI free) for 1 and 2 weeks. We then performed IC_50_ measurements, and evaluated their IRI-resistance level using the resistance index (RI), which is the ratio of IC_50_ of IRI-resistance and parental HCT116 cells (IC_50_ = 2.90 ± 0.1 μM).^49^ Our results indicate that their IRI-resistance levels were slightly reduced after 1 week (RI = 5.0) and 2 week (RI = 4.0) culture in the IRI-free medium, compared with those of the original IRI-resistant cells (RI = 5.7) (Fig. S1†).

### Change of drug-resistance levels of drug-sensitive cells under co-culture conditions

**Fig. 2:**
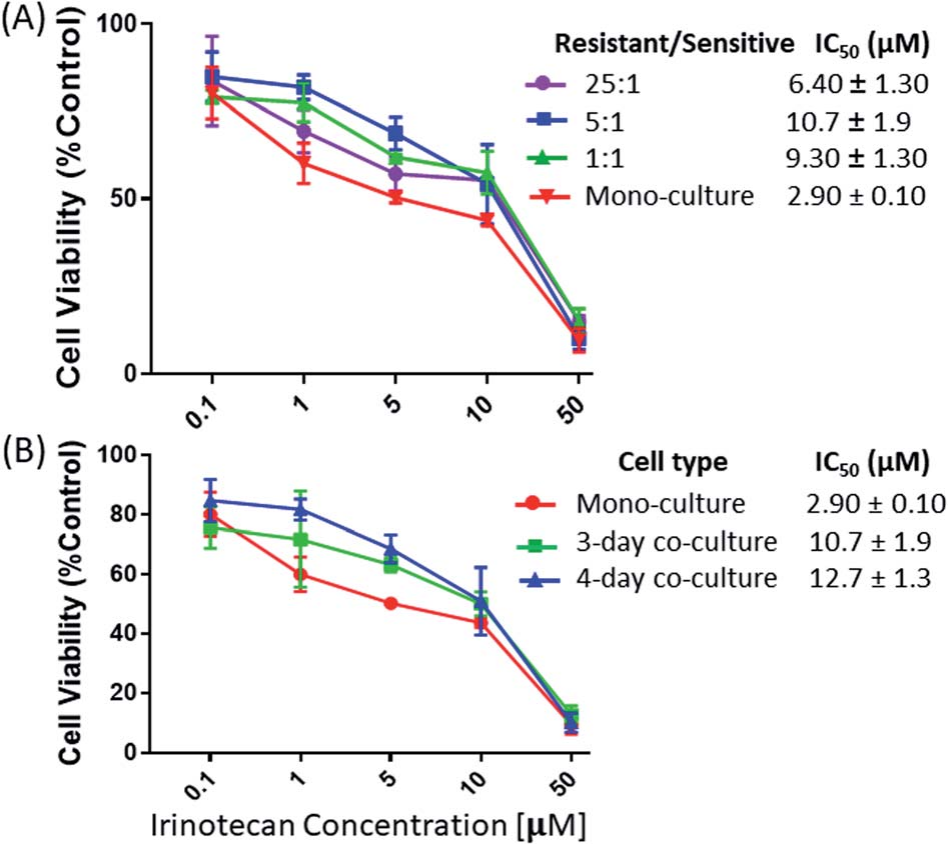
The viability measurements of drug-sensitive HCT116 cells in the indirect co-culture with IRI- HCT116 cells at (A) different cell density ratios and (B) different co-culture times. Cell viability was reported relative to control cells (mono-cultured HCT116 cells) from 5 measurements.

We studied the influence of drug-resistant cells on the resistance level of drug-sensitive cells in both indirect and direct co-culture systems. In the indirect co-culture system, MTT assay was used to measure viabilities of HCT116 cells. Cells were co-cultured using different cell density ratios and time lengths. To study the influence of the relative cell populations, the IRI-HCT116 and HCT116 cells with different ratios (1 : 1, 5 : 1, and 25 : 1) were added into a combined Corning Transwell set. MTT assay was then carried out to determine the drug resistance levels of HCT116 cells after 2, 3, and 4 days of co-culture. Our results indicate that the ratio of IRI-resistant to HCT116 cells has a negligible influence when it was increased from 1 : 1 (IC_50_ = 9.30 ± 1.30 μM) to 5 : 1 (IC_50_ = 10.7 ± 1.9 μM) (Fig. 2A). However, a noticeable decrease of the IRI-resistance (IC_50_ = 6.40 ± 1.30 μM) was observed when this ratio was increased to 25 : 1. Based on our observation using a microscope, this change is likely due to the formation of IRI-HCT116 spheroids, instead of maintaining the 2D monolayer under other conditions, from excessive numbers of IRI-resistant cells. The 3D structure of spheroids is expected to limit interactions between IRI-HCT116 cells inside spheroids and HCT116 cells. Only those on the outer layer, which account for small portions of total cell numbers, can more effectively participate in such interactions. The influence of the co-culture time on the IRI-resistance has also been investigated. Our results indicate that HCT116 (IC_50_ = 2.90 ± 0.10 μM) acquired significantly higher levels of drug resistance after being co-cultured for 3 (IC_50_ = 10.7 ± 1.9 μM) and 4 days (IC_50_ = 12.7 ± 1.3 μM) (Fig. 2B).

**Fig. 3:**
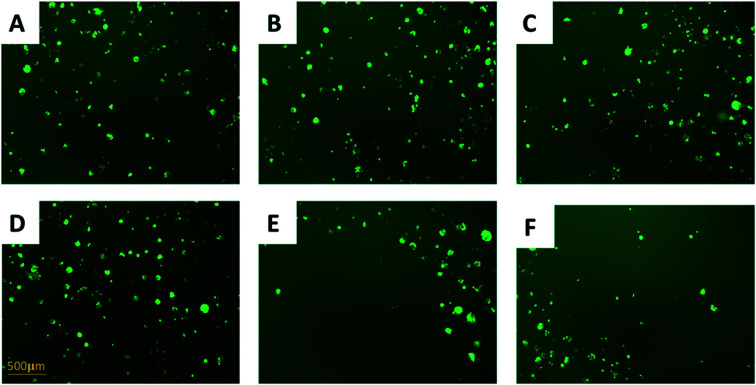
Using fluorescence microscopy (with a 10× objective lens) to evaluate drug resistance of HCT116-GFP cells in the direct co-culture with (A–C) drug-resistant or (D–F) drug-sensitive HCT116 cells, *λ*_excitation_ = 495 nm, and *λ*_emission_ = 519 nm. (A) Abundant HCT116-GFP cells (light-green spots) were observed in the co-culture system with IRI-HCT116 cells (no fluorescence). Densities of HCT116-GFP were slightly reduced after IRI treatment at (B) 5 μM and (C) 10 μM for 24 h. (D) Abundant HCT116-GFP cells were observed in the co-culture system with regular HCT116 cells. Densities of HCT116-GFP were significantly reduced after IRI treatment at (B) 5 μM and (C) 10 μM for 24 h.

In the direct co-culture systems, IC_50_ measurements of cells cannot be conveniently conducted. Instead, the cell density was visually inspected to approximately evaluate changes of the drug-resistance level under IRI treatment. The IRI-HCT116 and HCT116-GFP cells (with an initial cell density ratio of 1 : 1) were cultured in the same well containing IRI (0, 5.0, or 10 μM). We used a fluorescence microscope to monitor the growth of HCT116-GFP cells under IRI treatment. In the comparison studies, HCT116 and HCT116-GFP cells were co-cultured under the same conditions (Fig. 3). Fluorescence images of each system were taken after 24 h of treatment. It is evident that HCT116-GFP cells acquired higher levels of drug resistance through interactions with IRI-HCT116 cells (Fig. 3A–C), whereas those co-cultured with regular HCT116 (Fig. 3D–F) exhibited poorer viability under IRI treatment. In more quantitative analyses, the relative intensities of fluorescence (calculated using ImageJ) from HCT116-GFP cells were used to compare their viability at different IRI treatment concentrations (Fig. S2†), and we observed the similar trends. Our experimental results suggest that drug-resistant cells can foster drug-sensitive cells to improve their resistance levels through cell–cell interactions under both indirect and direct co-culture conditions.

### Metabolomics profile change of cells in co-culture systems

**Fig. 4:**
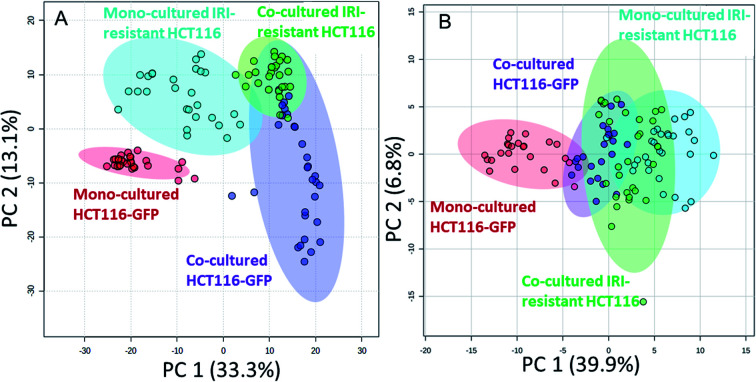
PCA results illustrating metabolomics profiles of HCT116-GFP and IRI-HCT116 cells in the mono-culture and direct co-culture systems. The results were obtained from both the (A) positive and (B) negative ion modes. Metabolites of HCT116-GFP were significantly changed by IRI-HCT116 cells in both systems, and these two types of cells tended to possess similar metabolomics profiles in the direct co-culture.

We performed the SCMS experiments for cells in five different groups, including the mono-cultured (HCT116, HCT116-GFP, and IRI-resistant HCT116) and co-cultured (HCT116-GFP and IRI-resistant HCT116) cells. Approximately 30 cells in each group were analyzed, and the obtained data were subjected to the pretreatment and statistical analyses to extract molecular information (Fig. 4). To minimize the variance of experimental conditions (*e.g.*, instrument tuning and cell status) in the SCMS results, we performed three batches of experiments on three different days.

In the first batch of the SCMS experiment, we compared the metabolomics profiles between HCT116 and HCT116-GFP cells in mono-culture, in order to verify that GFP labelling has no significant influence on cell metabolites in our experiments. We performed PCA, an unsupervised multivariate analysis method, and the results indicate that HCT116 and HCT116-GFP cells possess very similar molecular profiles (*i.e.*, ellipses of cells in these two groups are largely overlapped) (Fig. S3A and S3B†). To quantitatively confirm these results, we subjected these SCMS data to PLS-DA, a supervised method, and the results show that there are no significantly different (*p* = 0.698) molecular compositions between these two cell lines (Fig. S3C†). Our experimental results agree well with the previous report that GFP labelling has a negligible influence on cell metabolism.^51^ Therefore, it is valid to use HCT116-GFP cells to represent HCT116 cells for the co-culture studies.

In the second batch of SCMS experiments, we studied IRI-resistant and HCT116-GFP cells in the direct co-culture, along with the mono-culture counterparts in both positive and negative ion modes. The PCA results from the positive ion mode indicate that the metabolic profiles of HCT116-GFP cells were significantly changed by the IRI-HCT116 cells through cell–cell interactions (Fig. 4A). Interestingly, both HCT116-GFP and IRI-resistant cells tend to exhibit increased similarities of metabolomics profiles after direct co-culture.

**Fig. 5:**
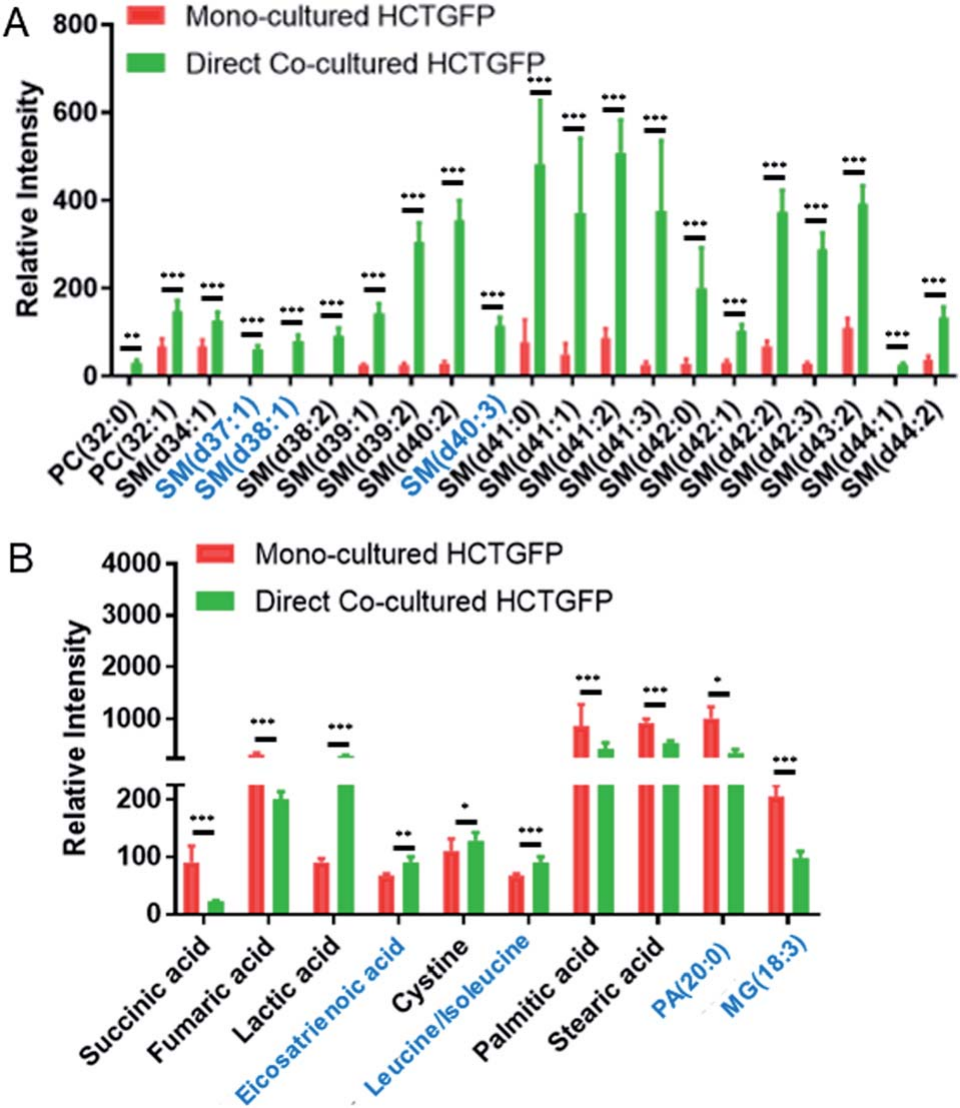
Metabolites significantly altered in single HCT116-GFP cells through direct co-culture with IRI-HCT116 cells. The results were obtained from the (A) positive and (B) negative ion modes, respectively, using *n* > 30 cells in each group. Species in the black font were identified from MS/MS analyses. SM (sphingomyelin), PC (phosphatidylcholine), PA (phosphatidic acid), and MG (monoglyceride). (From the *t*-test: *p < 0.05; **p < 0.01; ***p < 0.005).

To investigate molecules altered by co-culture, we compared the SCMS data of HCT116-GFP cells in the mono-culture and direct co-culture. Typical background-subtracted mass spectra of single HCT116-GFP cells under these two culture conditions are shown in Fig. S4,† illustrating the overall cellular metabolites of drug-sensitive cells affected by drug-resistant cells. In general, HCT116-GFP cells from direct co-culture contain higher abundances of SMs (*e.g.*, SM(40:1) and SM(41:1)) than triglycerides (TGs) (*e.g.*, TG(52:2) and TG(54:3)), whereas an opposite trend was observed in mono-cultured HCT116-GFP cells. The Student's *t*-test was further performed to discover species altered by co-culture. 19 sphingomyelins (SMs) and 2 phosphatidylcholines (PCs) were significantly upregulated in HCT116-GFP cells from direct co-culture (Fig. 5A).

To improve the detection coverage of cellular metabolites, we conducted the third batch of SCMS experiments in the negative ion mode. HCT116-GFP and IRI-resistant cells in both the direct co-culture and mono-culture were analyzed. Similar to the trend observed from the positive ion mode results, metabolites detected in the negative ion mode indicated that HCT116-GFP and IRI-resistant cells tend to exhibit increased similarities (Fig. 4B). The overlap degrees among different cell groups are different in two ion modes, likely because fewer metabolites were observed in the negative ion mode (Fig. S5†).

The *t*-test was conducted to determine metabolites with significant changes between mono-cultured and co-cultured HCT116-GFP cells. As illustrated in Fig. 5B, the top-10 metabolites with significantly different abundances include amino acids, fatty acids, and species involved in the TCA (tricarboxylic acid) cycle. The current study focuses on the influence of drug-resistant cells on drug-sensitive cells through cell-to-cell interactions. Nevertheless, the comparison of the relative abundances of metabolites among other cell groups were also performed (Fig. S6†). In general, co-cultured cells tend to possess metabolites with similar abundances as reflected in Fig. 4. Future studies are needed to investigate their metabolomics pathways relevant to drug-resistance.

To study the influence of drug treatment on cell metabolites, SCMS experiments were conducted using HCT116-GFP and IRI-resistant cells with and without IRI treatment (Fig. S7†). As expected, drug treatment has more influence on drug-sensitive cells than drug-resistant cells (Fig. S7A†). The *t*-test results indicate that species altered by IRI treatment in HCT116-GFP cells and IRI-resistant cells are primarily phospholipids (*e.g.*, PC, PE, and LysoPC lipids), which agree with previous studies.^47,60,61^ Our results suggest that molecules changed under co-culture conditions are primarily due to cell-to-cell interactions rather than the influence of the drug compound. The structural identification was performed using MS/MS both at the single-cell level and using cell lysate (Fig. S8–S11†).

### Potential metabolic mechanisms of spreading drug resistance by drug-resistant cells

The tumor microenvironment, composed of different types of cells, plays an important role in spreading drug resistance, resulting in cancer relapse and metastasis.^62^ Growing evidence shows that the metabolic cooperation between different types of cells promotes the development of drug resistance.^63,64^ In particular, cell communication between drug-resistant cells and drug-sensitive cells can assist the progression of chemotherapy resistance in tumor cells.^10^ The attainment of drug resistance in drug-sensitive cells from drug-resistant cells can be achieved by exchanging signaling factors.^10,12,13,65^ For example, drug resistance levels of drug-sensitive cells can be enhanced by intercellular transfer of P-glycoprotein (P-gp) from drug-resistant cells.^11,66–69^ The acquirement of these mediators elevates drug resistance, and subsequently induces metabolic regulation in drug-sensitive cells.^70^

In the current studies, we detected lipid reprograming in drug-sensitive cells after acquiring drug resistance from their neighboring drug-resistant cells. Lipids are a major constituent of the cell membrane, which is the signaling platform of transporters, and they control the structure and permeability of the membrane. Our positive ion mode results indicate that compared with the mono-cultured HCT116-GFP cells, the major alteration of lipids in co-cultured HCT116-GFP cells is attributed to significantly upregulated SMs (Fig. 5A). Similar results have been reported in previous studies, with higher levels of SM lipids being associated with drug resistance in many cancer resistant cell lines (*e.g.*, 5-FU-resistant human colorectal cancer cells,^71^ doxorubicin-resistant ovarium carcinoma cells,^72^ and vinblastine-resistant leukemia T lymphoblast cells^73^). Particularly, the increased levels of SM in drug-resistant cells can alter biophysical properties of the cell membrane by increasing its structural order and decreasing its fluidity, possibly due to SMs' high affinities to cholesterol.^74^ Passive diffusion of drugs across the cell membrane, which is one of the most efficient drug uptake methods, is thus impaired by the decreased membrane fluidity.^74^ This mechanism of biophysical alternation on the cell membrane could possibly explain the elevated SM expression in co-cultured HCT116-GFP cells in our study. However, more experiments need to be conducted to verify the proposed mechanism.

Additional information can be obtained from the negative ion mode results (Fig. 5A). It is possible that cell–cell communication can also induce drug resistance through other pathways such as the TCA cycle, amino acid metabolism, and fatty acid expression. For example, succinic acid and fumaric acid, two small molecules in TCA cycle metabolism, were significantly downregulated in HCT116-GFP cells after co-culturing with IRI-resistant cells. The TCA cycle is known to play a central role in the development of drug resistance.^75^ Our findings are consistent with previous studies: genes involved in the TCA cycle are significantly downregulated in the IRI-resistant cells compared to those in the sensitive cells.^76^ Succinic acid and fumaric acid are also classified as oncometabolites, and their downregulation is tightly correlated with the development of drug resistance in cisplatin-resistant cells.^77^ The suppressed TCA cycle impels cancer cells to rely more on glycolysis for obtaining energy, thus producing larger amounts of lactic acid (Fig. 5B). In fact, accumulation of lactic acid is related to increased cancer resistance and malignancy.^78^ Amino acid metabolism is also associated with the development of drug resistance in cancer. For example, the overexpression of the cystine transport system assists cancer cells in decreasing drug sensitivity.^79^

## Conclusions

We provided a new method to study cellular metabolism alteration due to direct cell–cell interactions among different types of cells by combining the Single-probe SCMS and fluorescence microscopy. Our method allows for ambient SCMS metabolomics studies of different types of live cells growing under the same conditions with direct cell–cell contact. We used model systems representing drug-sensitive (*i.e.*, parental HCT116 or HCT116-GFP) and drug-resistant (*i.e.*, IRI-resistant HCT116) cells in both indirect (without cell–cell contact) and direct (with cell–cell contact) co-culture systems. This study is focused on the influence of drug-resistant cells on the drug-resistance level and metabolism of drug-sensitive cells under co-culture conditions. A fluorescence microscope was used to locate individual HCT116-GFP cells in the direct co-culture system, and then the Single-probe SCMS technique was used to measure cellular metabolites. Our results indicate that drug-sensitive cancer cells acquired significantly improved drug-resistance levels and drastically altered metabolomics profiles through cell–cell communication with drug-resistant cancer cells. In particular, the drug-sensitive cancer cells tend to acquire metabolites similar to those of drug-resistant cells through direct co-culture. The acquirement of enhanced drug resistance is associated with multiple metabolism regulations such as higher expression of SM lipids and lactic acid and downregulation of TCA cycle intermediates. Detailed structural characterization of lipids (*e.g.*, the determination of cis/trans isomers and positions of carbon–carbon double bonds) was not performed, because these studies are beyond our current research scope. Future studies are necessary to fill this knowledge gap. We expect that our methods can be potentially utilized to study metabolic responses of single cells to microenvironment change (*e.g.*, physical and chemical stimuli) or cell–cell interactions using cells labelled with fluorescence proteins or dyes.

## Data availability

Experimental MS data, including SCMS, LC-MS/MS, and direct injection MS/MS, have been deposited in MassIVE (accession number MSV000089496).

## Author contributions

Z. Yang and X. Chen designed the studies. X. Chen conducted the experiments and data analysis. Z. Kai assisted in data collection of cell viability measurement and SCMS experiments. The initial draft was prepared by X. Chen. Z. Yang supervised the project and reviewed/edited the manuscript.

## Conflicts of interest

The authors declare no competing financial interest.

## Supplementary Material

SC-013-D2SC02298B-s001
