# Supplementary material for: Metabolomics studies of cell–cell interactions using single cell mass spectrometry combined with fluorescence microscopy
Source: Chem Sci. 2022 May 16;13(22):6687–95. doi: 10.1039/d2sc02298b (PMC9172575; doi:10.1039/d2sc02298b)
Supplement: SC-013-D2SC02298B-s001 [file SC-013-D2SC02298B-s001.pdf]

Electronic Supplementary Material (ESI) for Chemical Science.
This journal is © The Royal Society of Chemistry 2022

Supporting Information

Metabolomics Studies of Cell-Cell Interactions using Single Cell Mass Spectrometry Combined with Fluorescence Microscopy

Xingxiu Chen,[a] Zongkai Peng,[a] and Zhibo Yang*[a]

[a] Department of Chemistry and Biochemistry, University of Oklahoma, Norman, Oklahoma 73019, USA

Table of Contents

Supplementary Figures 2

Fig. S1. Cell viability mesurements 2

Fig. S2. ImageJ quantification of fluorescence intensity 3

Fig. S3. PCA and PLS-DA plots. 4

Fig. S4. Background-subtracted SCMS spectra (positive ion mode) 5

Fig. S5. Background-subtracted SCMS spectra (negative ion mode) 6

Fig. S6. T-test results of mono-culture and co-culture experiments 7

Fig. S7. PCA and t-test results of IRI treatment experiment. 8

Fig. S8. MS^2 at the single-cell level 9

Fig. S9. MS^2 of cell lysate using direction injection 13

Fig. S10. MS^2 of cell lysate using HPLC-MS 15

Fig. S11. MS^2 of cell lysate in the negative ion mode 18

Supplementary Figures

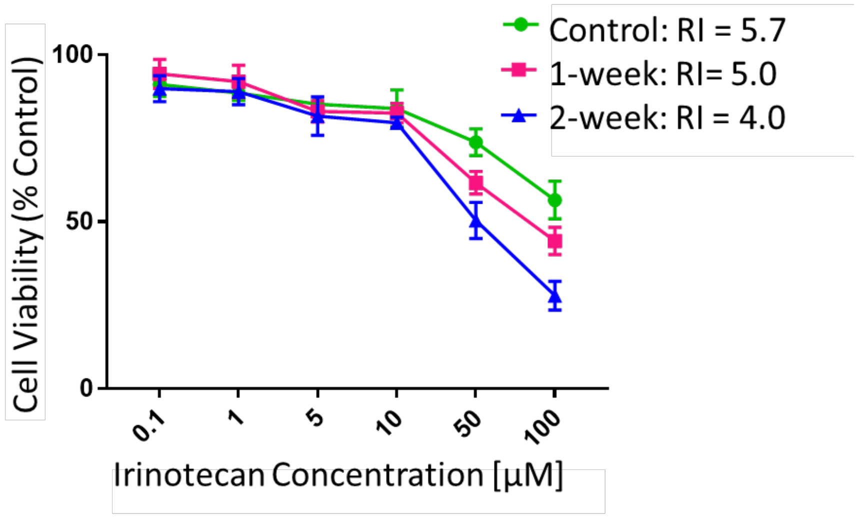

Fig. S1. Cell viability measurements (means ± SD) of IRI-HCT116 cells (Control) and IRI-HCT116 cells cultured in drug-free medium for 1 (1-week) or 2 weeks (2-week). RI (resistant index) was calculated as the ratio of IC_{50} of IRI-HCT116 cells under different conditions to that of the parental HCT116 cells. Data are obtained from n = 5 measurements.

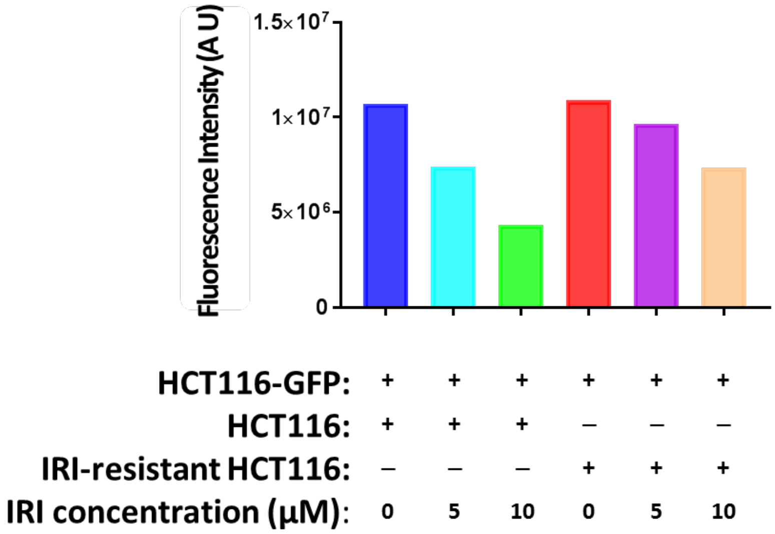

Fig. S2. Quantification of fluorescence intensity of HCT116-GFP cells in the co-culture systems under different IRI treatment concentrations. HCT116-GFP cells co-cultured with the regular HCT116 cells exhibited lower fluorescence intensity (i.e., lower proliferation) compared with those co-cultured with IRI-HCT116 cells. Fluorescence images were captured using a 4X objective lens to include all cells in a well. Fluorescence intensities of fluorescence images (n = 3 replicates) were quantified using ImageJ.

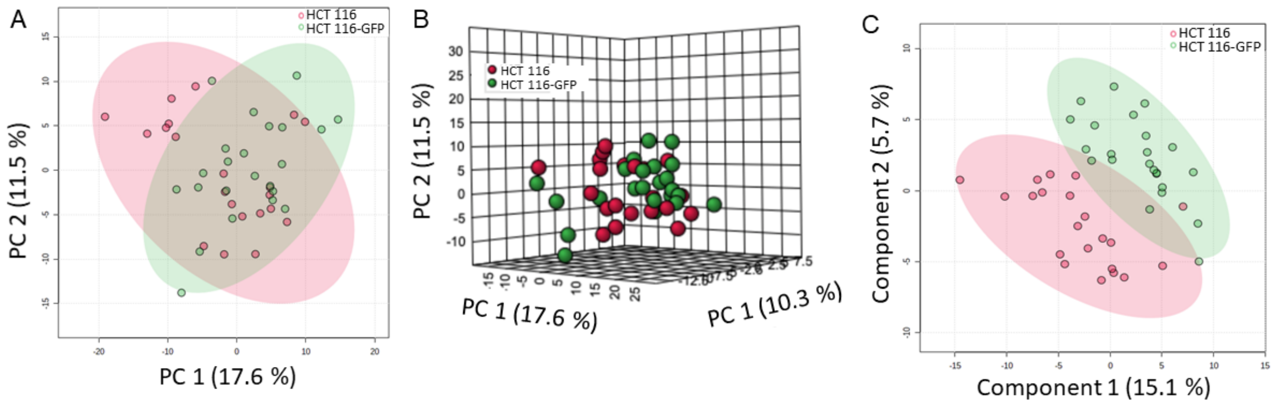

Fig. S3. Comparison of SCMS metabolomics profiles of mono-cultured HCT116 and HCT116-GFP cells using (A) 2D PCA, (B) 3D PCA, and (C) 2D PLS-DA (n = 20 in each group). Both PCA and PLS-DA (p = 0.698) results show that there is no significant difference of metabolic profiles between these two types of cells.

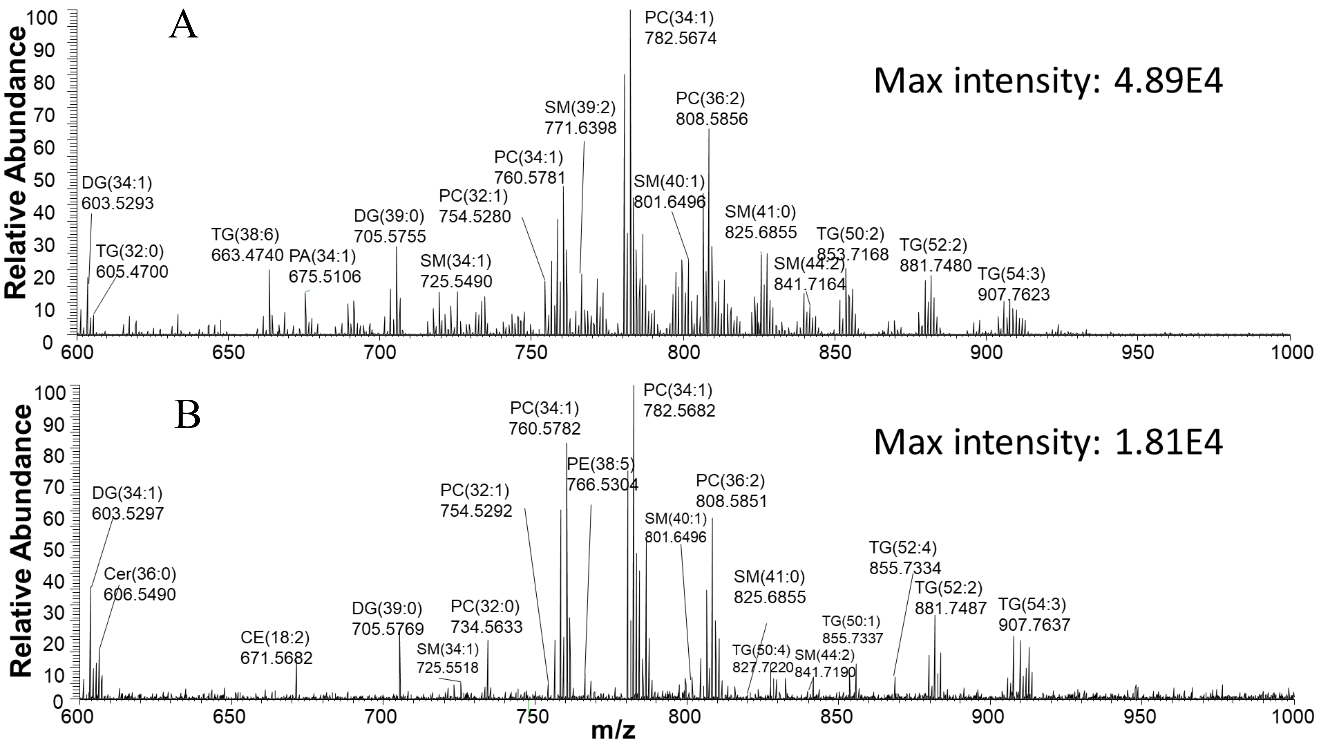

Fig. S4. Typical background-subtracted SCMS spectra of (A) a co-cultured HCT116-GFP cell and (B) a mono-cultured HCT116-GFP cell in the positive ion mode. Species are tenatively labeled by searching the measured m/z values through online databases. The range of m/z = 600–1,000 was illustrated to compare different SMs expression levels.

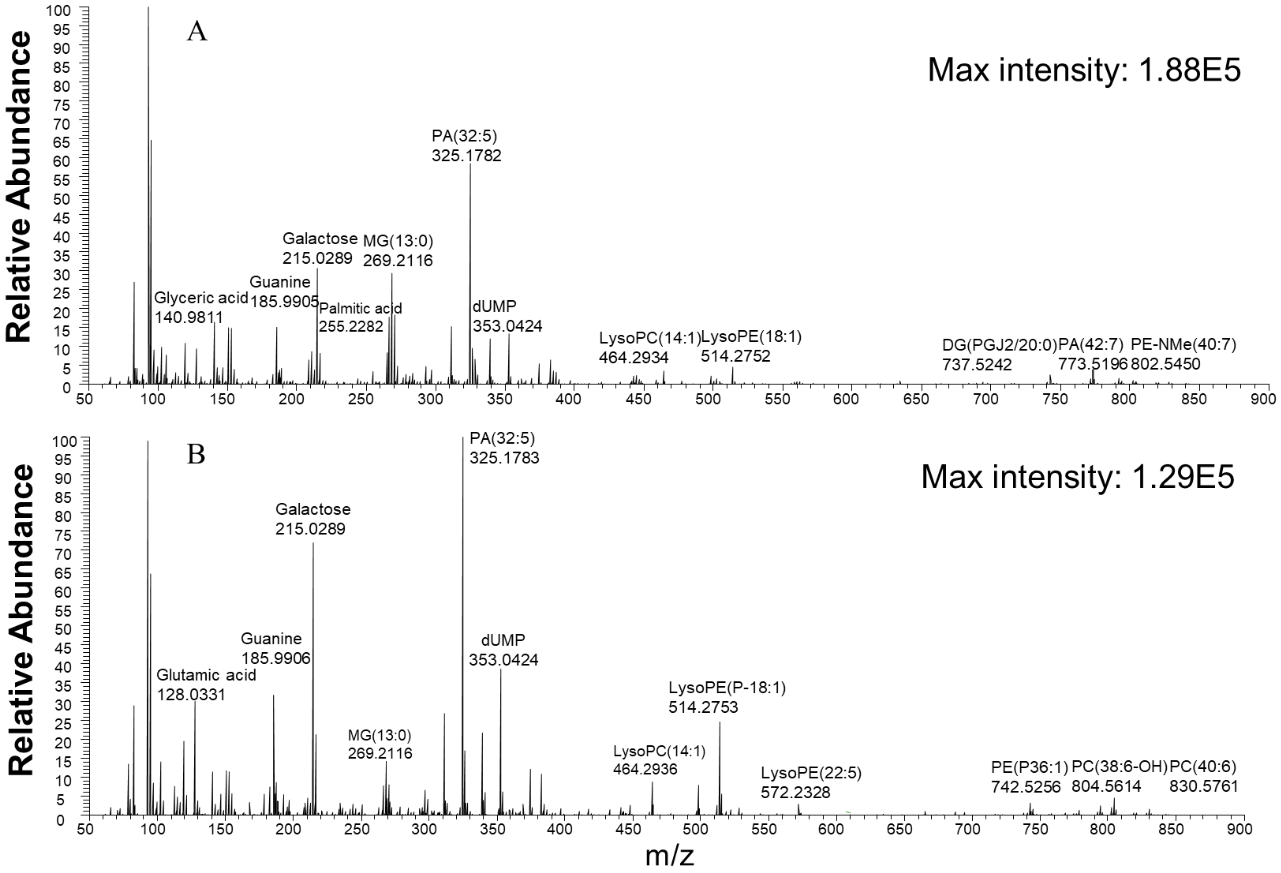

Fig. S5. Typical background-subtracted SCMS spectra of (A) a co-cultured HCT116-GFP cell and (B) a mono-cultured HCT116-GFP cell in the negative ion mode. Species are tenatively labeled by searching the measured m/z values through online databases.

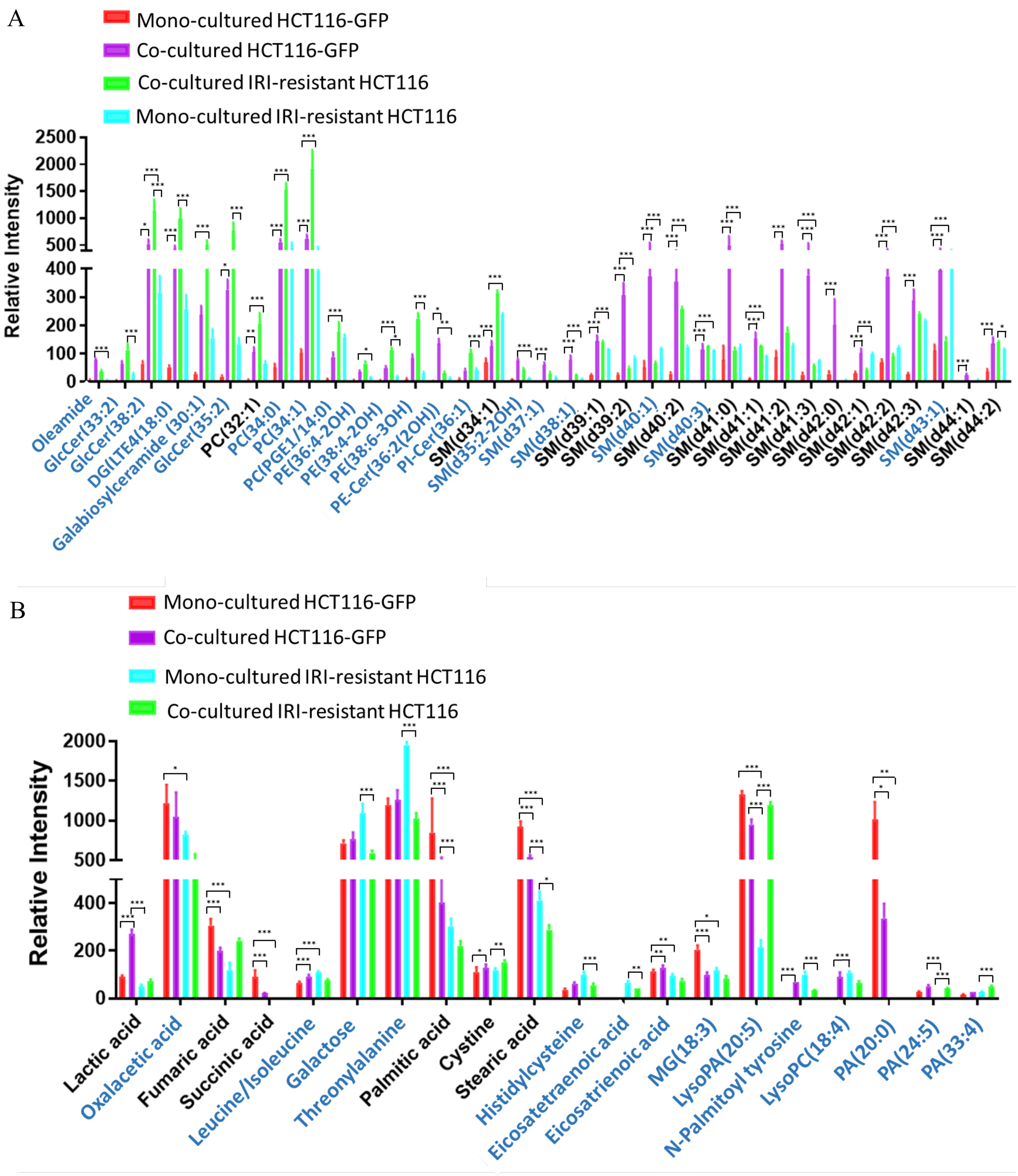

Fig. S6. Comparison of the relative abundances of metabolites in cells from different groups. Results were obtained from (A) the positive and (B) negative ion modes. T-test was used for the comparison of mono-cultured HCT116-GFP and mono-cultured IRI-resistant HCT116, mono-cultured IRI-resistant HCT116 and co-cultured HCT116-GFP, and co-cultured IRI-resistant HCT116 and mono-cultured IRI-resistant HCT116 cells. Species in black font were identified from MS^2 analyses. (From t-test: * p<0.05, **p<0.01, ***p<0.005.)

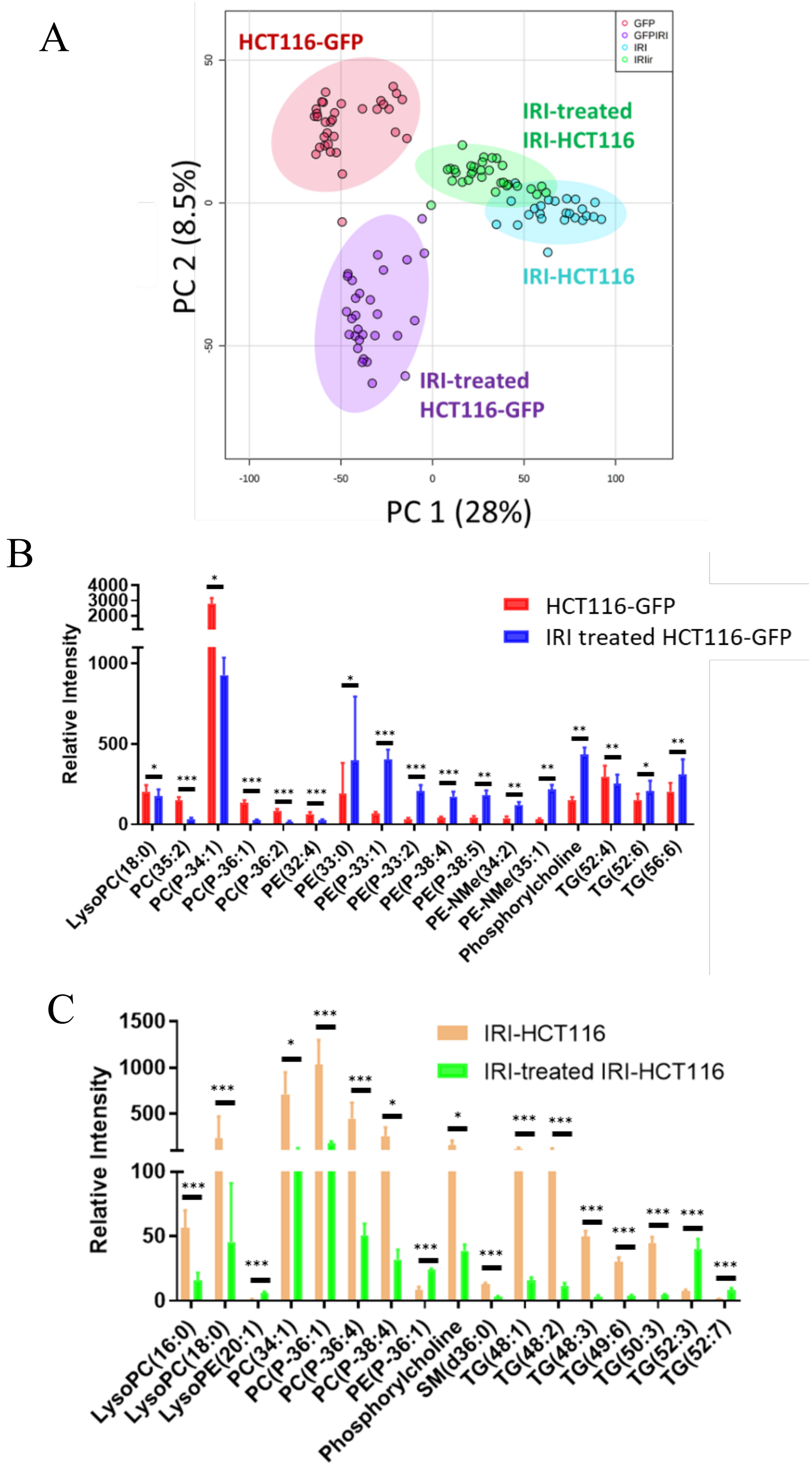

Fig. S7. Influence of IRI treatment on the metablomic profiles of single HCT116-GFP (drug-sensitive) and IRI-HCT116 (drug-resistant) cells from mono-culture. (A) PCA results of cells with and without drug treatment. T-test results showing species with significantly altered abundances in (B) HCT116-GFP and (C) IRI-HCT 116. IRI at IC_{50} (2.9 μM and 16 μM for HCT116-GFP and IRI-resistant cells, respectively) was used for treatment. Experiments were conducted in the positive ion mode (n = 30 in each group). Species were tenatively labeled. (From t-test: * p<0.05, **p<0.01, ***p<0.005.)

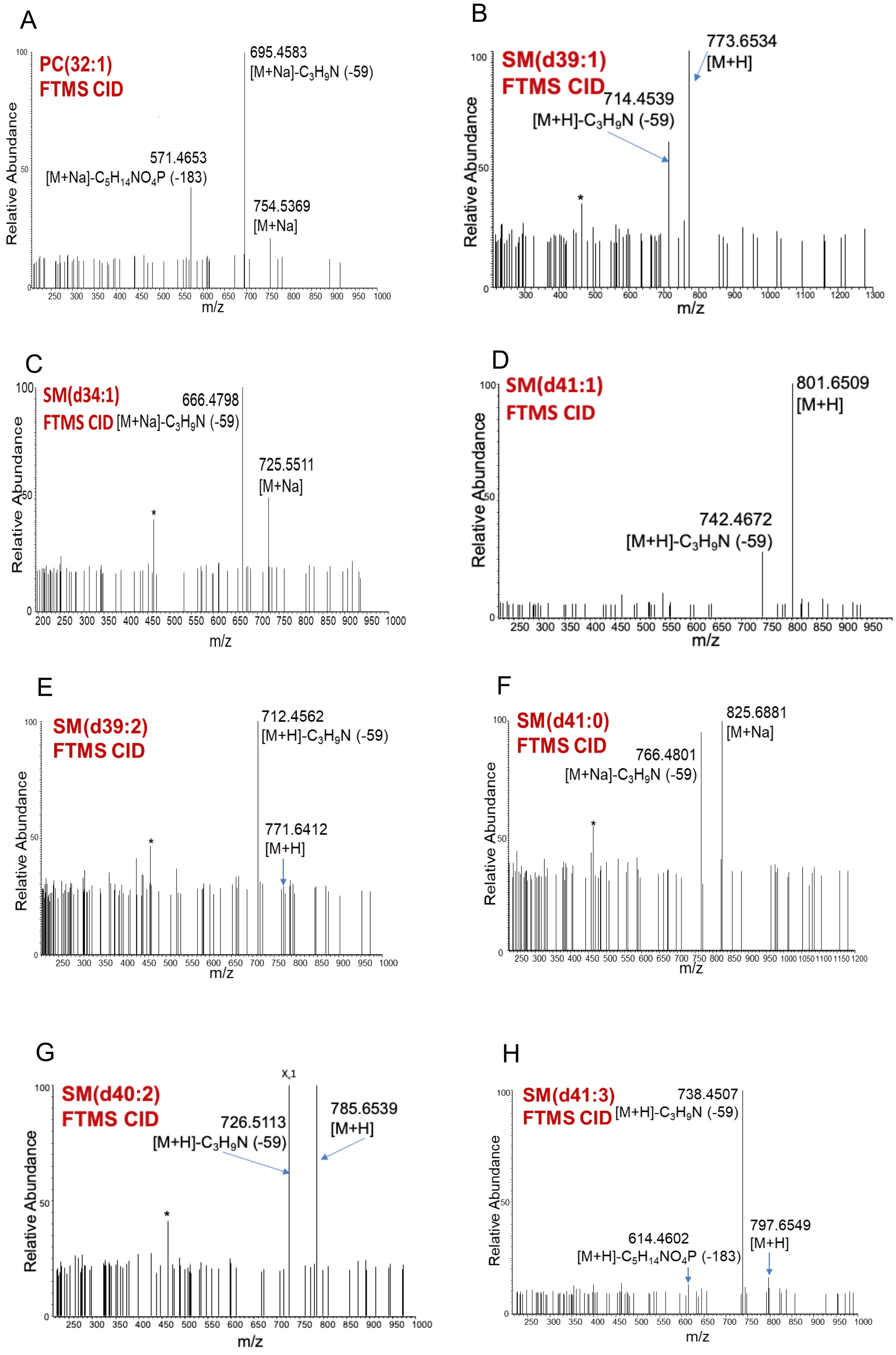

A
PC(32:1)
FTMS CID
695.4583
[M+Na]-C3H9N (-59)
571.4653
[M+Na]-C5H14NO4P (-183)
754.5369
[M+Na]
Relative Abundance
m/z
B
SM(d39:1)
FTMS CID
773.6534
[M+H]
714.4539
[M+H]-C3H9N (-59)
Relative Abundance
m/z
C
SM(d34:1)
FTMS CID
666.4798
[M+Na]-C3H9N (-59)
725.5511
[M+Na]
Relative Abundance
m/z
D
SM(d41:1)
FTMS CID
801.6509
[M+H]
742.4672
[M+H]-C3H9N (-59)
Relative Abundance
m/z
E
SM(d39:2)
FTMS CID
712.4562
[M+H]-C3H9N (-59)
771.6412
[M+H]
Relative Abundance
m/z
F
SM(d41:0)
FTMS CID
825.6881
[M+Na]
766.4801
[M+Na]-C3H9N (-59)
Relative Abundance
m/z
G
SM(d40:2)
FTMS CID
726.5113
[M+H]-C3H9N (-59)
785.6539
[M+H]
Relative Abundance
m/z
H
SM(d41:3)
FTMS CID
738.4507
[M+H]-C3H9N (-59)
614.4602
[M+H]-C5H14NO4P (-183)
797.6549
[M+H]
Relative Abundance
m/z

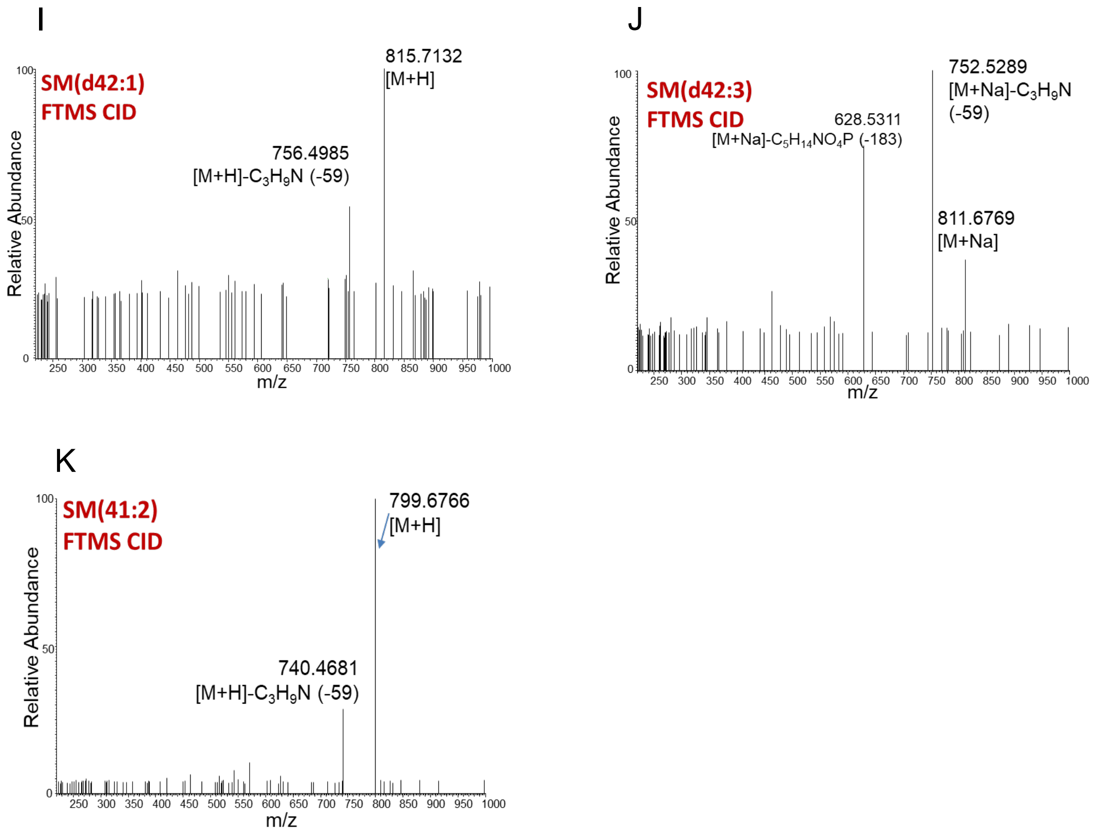

Fig. S8A. MS^2 identification of ions of interest at the single-cell level using collision-induced dissociation (CID) with Orbitrap mass analyzer (FTMS). (A) PC(32:1), (B) SM(d39:1), (C) SM(d34:1), (D) SM(d41:1), (E) SM(d39:2), (F) SM(d41:0), (G) SM(d40:2), (H) SM(d41:3), (I) SM(d42:1), (J) SM(d42:3), (K) SM(d41:2).

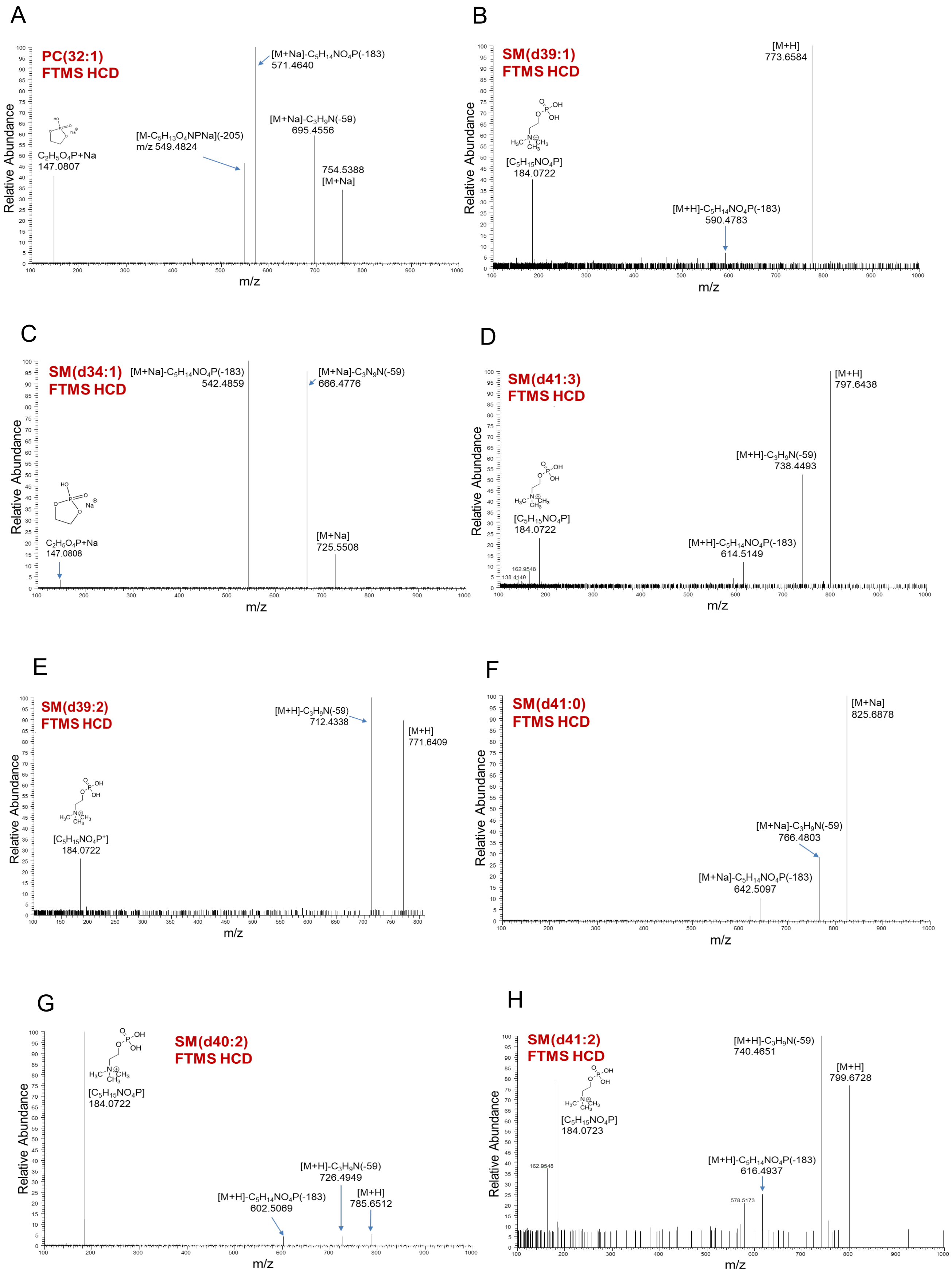
A
PC(32:1)
FTMS HCD
[M+Na]-C5H14NO4P(-183)
571.4640
[M+Na]-C3H9N(-59)
695.4556
[M-C5H13O4NPNa](-205)
m/z 549.4824
C2H5O4P+Na
147.0807
754.5388
[M+Na]
Relative Abundance
m/z
B
SM(d39:1)
FTMS HCD
[M+H]
773.6584
[C5H15NO4P]
184.0722
[M+H]-C5H14NO4P(-183)
590.4783
Relative Abundance
m/z
C
SM(d34:1)
FTMS HCD
[M+Na]-C5H14NO4P(-183)
542.4859
[M+Na]-C3N9N(-59)
666.4776
[M+Na]
725.5508
C2H5O4P+Na
147.0808
Relative Abundance
m/z
D
SM(d41:3)
FTMS HCD
[M+H]
797.6438
[M+H]-C3H9N(-59)
738.4493
[C5H15NO4P]
184.0722
[M+H]-C5H14NO4P(-183)
614.5149
162.9548
138.4149
Relative Abundance
m/z
E
SM(d39:2)
FTMS HCD
[M+H]-C3H9N(-59)
712.4338
[M+H]
771.6409
[C5H15NO4P+]
184.0722
Relative Abundance
m/z
F
SM(d41:0)
FTMS HCD
[M+Na]
825.6878
[M+Na]-C3H9N(-59)
766.4803
[M+Na]-C5H14NO4P(-183)
642.5097
Relative Abundance
m/z
G
SM(d40:2)
FTMS HCD
[C5H15NO4P]
184.0722
[M+H]-C3H9N(-59)
726.4949
[M+H]-C5H14NO4P(-183)
602.5069
[M+H]
785.6512
Relative Abundance
m/z
H
SM(d41:2)
FTMS HCD
[M+H]-C3H9N(-59)
740.4651
[M+H]
799.6728
[C5H15NO4P]
184.0723
[M+H]-C5H14NO4P(-183)
616.4937
162.9548
578.5173
Relative Abundance
m/z

I

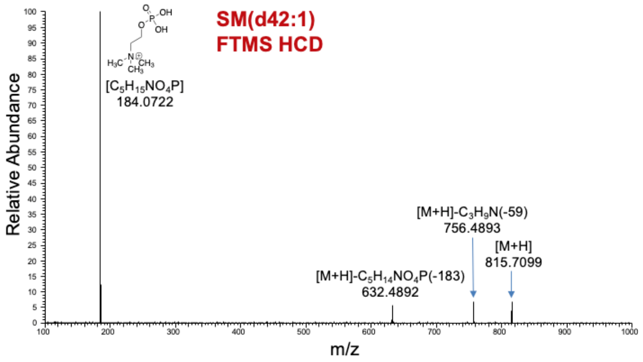

J

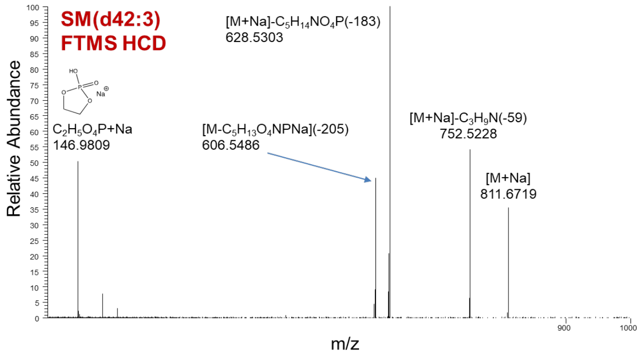

K

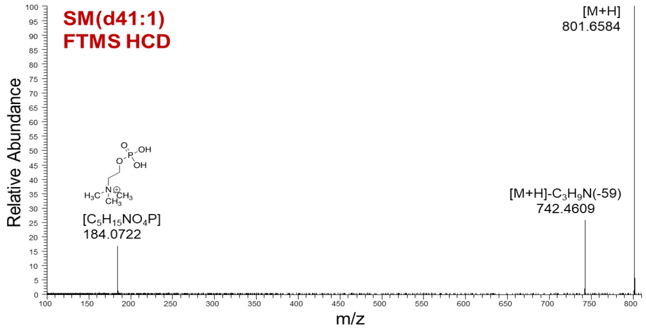

Fig. S8B. MS^2 identification of ions of interest at the single-cell level using higher energy collision dissociation (HCD) with Orbitrap mass analyzer (FTMS). (A) PC(32:1), (B) SM(d39:1), (C) SM(d34:1), (D) SM(d41:3), (E) SM(d39:2), (F) SM(d41:0), (G) SM(d40:2), (H) SM(d41:2), (I) SM(d42:1), (J) SM(d42:3), and (K) SM(d41:1).

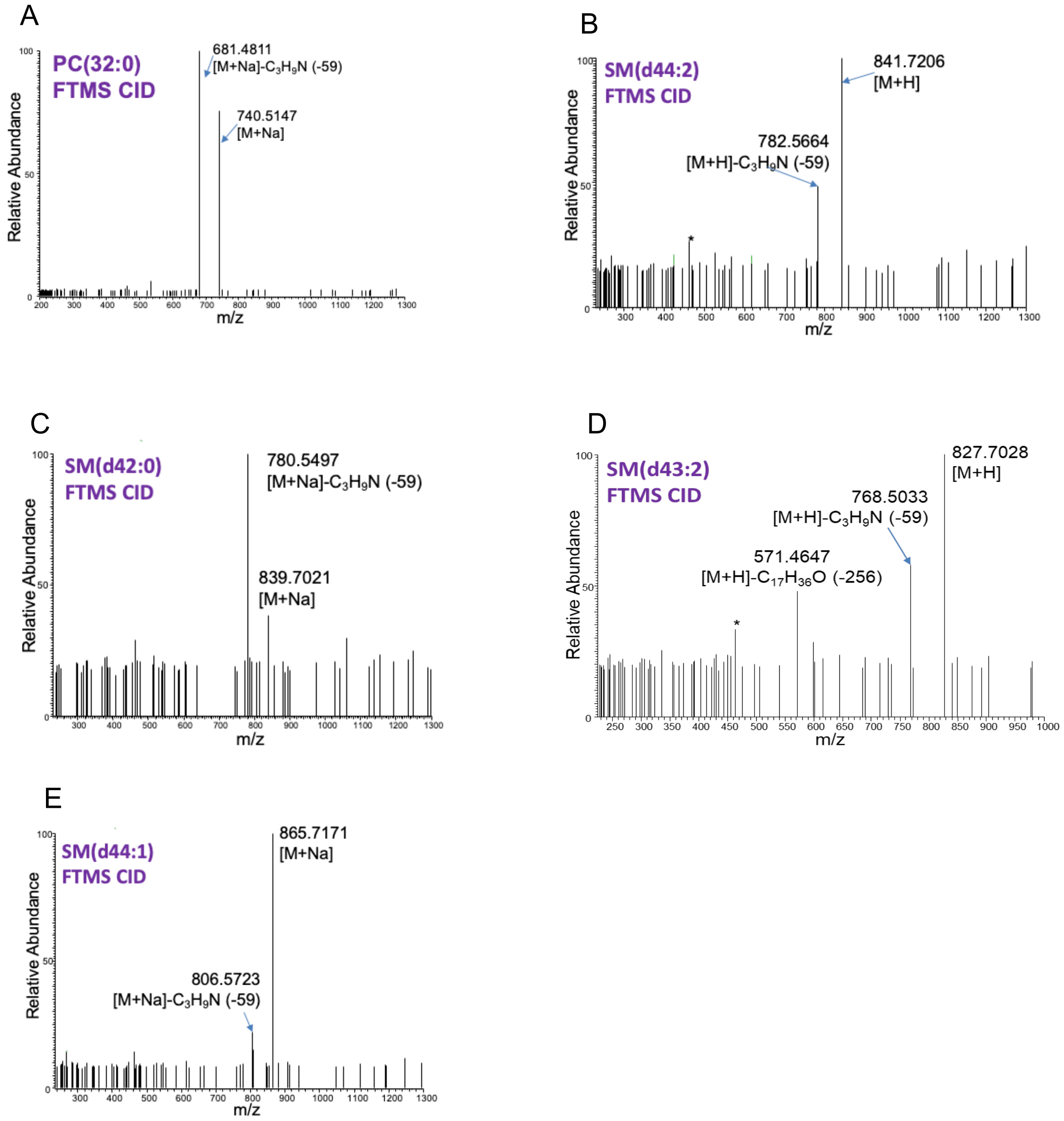

Fig. S9A. MS[2] identification of ions of interest from cell lysate using collision-induced dissociation (CID) with Orbitrap mass analyzer (FTMS). (A) PC(32:0), (B) SM(d44:2), (C) SM(d42:0), (D) SM(d43:2), and (E) SM(d44:1).

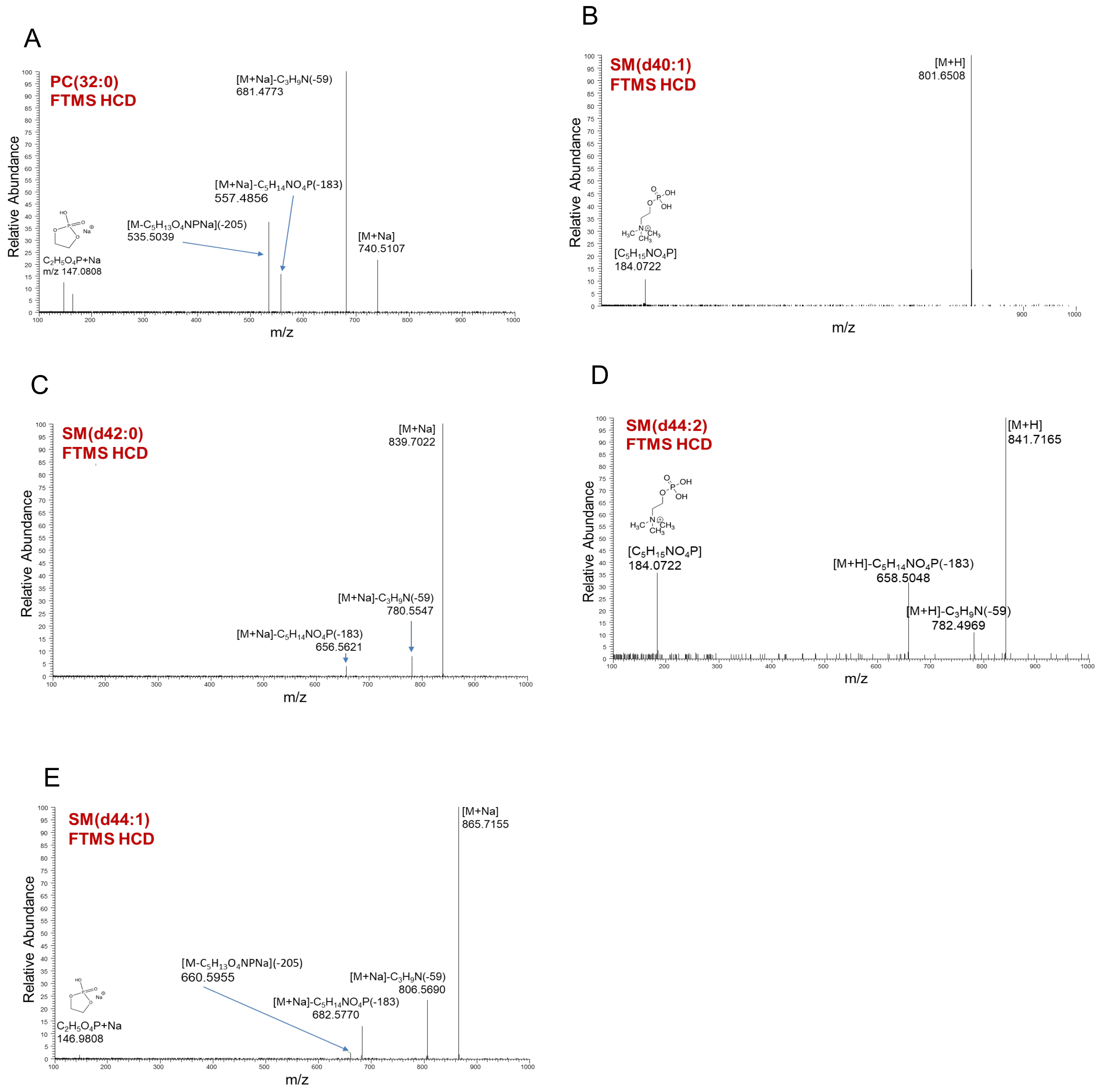

Fig. S9B. MS^2 identification of ions of interest from cell lysate (via direct injection) using higher energy collision dissociation (HCD) with Orbitrap mass analyzer (FTMS). (A) PC(32:0), (B) SM(d40:1), (C) SM(d42:0), (D) SM(d44:2), and (E) SM(d44:1).

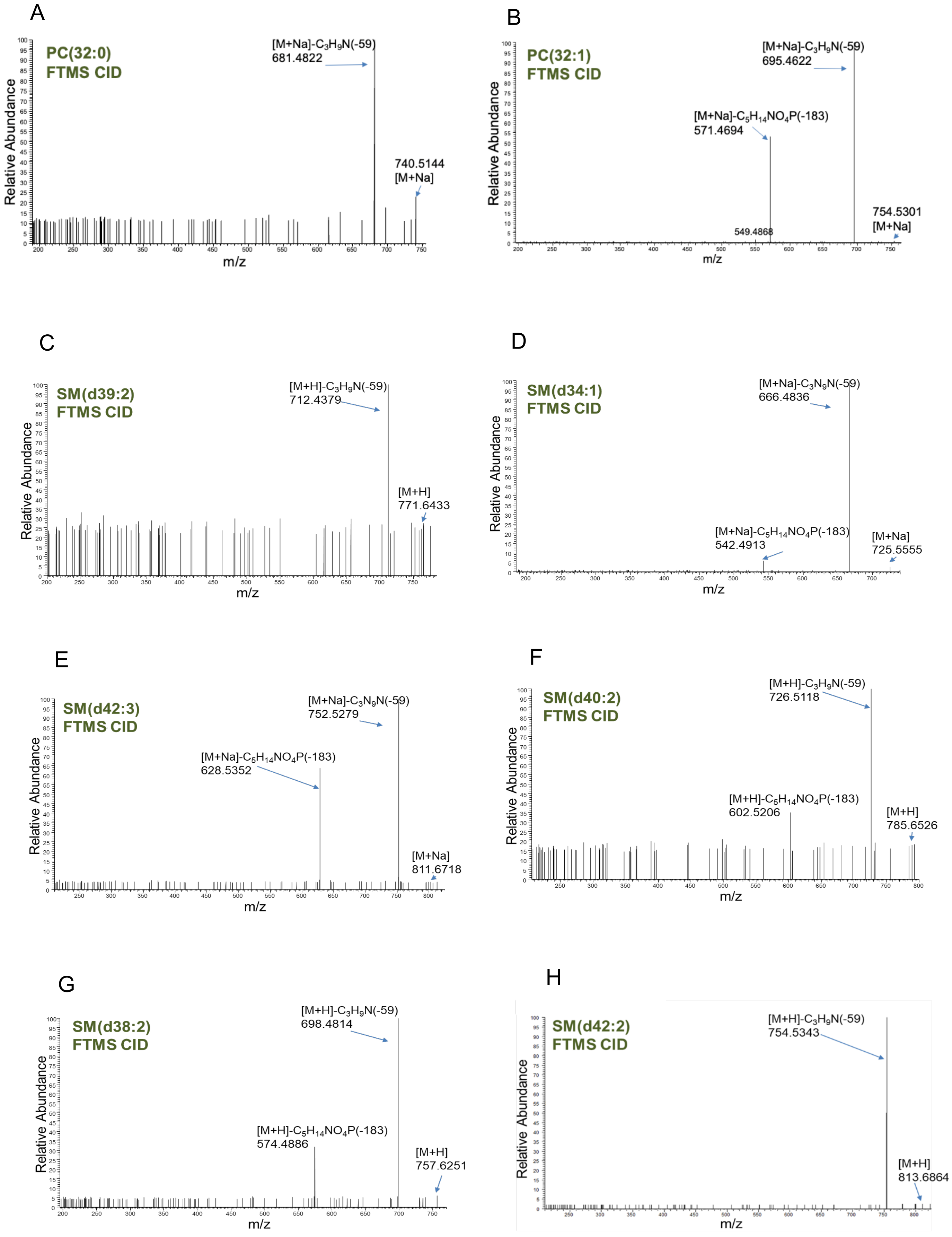
A
PC(32:0)
FTMS CID
[M+Na]-C_3H_9N(-59)
681.4822
740.5144
[M+Na]
Relative Abundance
m/z
B
PC(32:1)
FTMS CID
[M+Na]-C_3H_9N(-59)
695.4622
[M+Na]-$C_5H_{14}NO_4P$(-183)
571.4694
549.4868
754.5301
[M+Na]
Relative Abundance
m/z
C
SM(d39:2)
FTMS CID
[M+H]-C_3H_9N(-59)
712.4379
[M+H]
771.6433
Relative Abundance
m/z
D
SM(d34:1)
FTMS CID
[M+Na]-C_3N_9N(-59)
666.4836
[M+Na]-$C_5H_{14}NO_4P$(-183)
542.4913
[M+Na]
725.5555
Relative Abundance
m/z
E
SM(d42:3)
FTMS CID
[M+Na]-C_3N_9N(-59)
752.5279
[M+Na]-$C_5H_{14}NO_4P$(-183)
628.5352
[M+Na]
811.6718
Relative Abundance
m/z
F
SM(d40:2)
FTMS CID
[M+H]-C_3H_9N(-59)
726.5118
[M+H]-$C_5H_{14}NO_4P$(-183)
602.5206
[M+H]
785.6526
Relative Abundance
m/z
G
SM(d38:2)
FTMS CID
[M+H]-C_3H_9N(-59)
698.4814
[M+H]-$C_5H_{14}NO_4P$(-183)
574.4886
[M+H]
757.6251
Relative Abundance
m/z
H
SM(d42:2)
FTMS CID
[M+H]-C_3H_9N(-59)
754.5343
[M+H]
813.6864
Relative Abundance
m/z

I

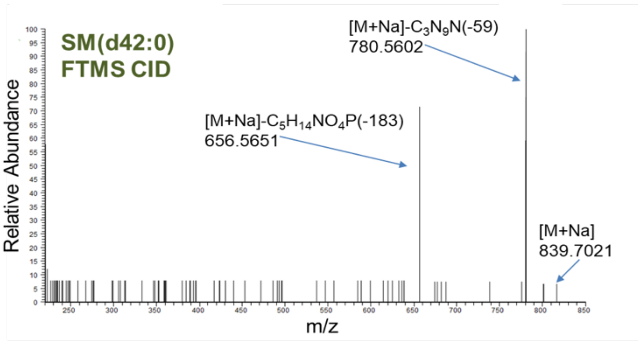

Fig. S10A. MS^2 identification of ions of interest from cell lysate (via HPLC-MS/MS) using collision-induced dissociation (CID) with Orbitrap mass analyzer (FTMS). (A) PC(32:0), (B) PC(32:1), (C) SM(d39:2), (D) SM(d34:1), (E) SM(d42:3), (F) SM(d40:2), (G) SM(d38:2), (H) SM(d42:2), (I) SM(d42:0).

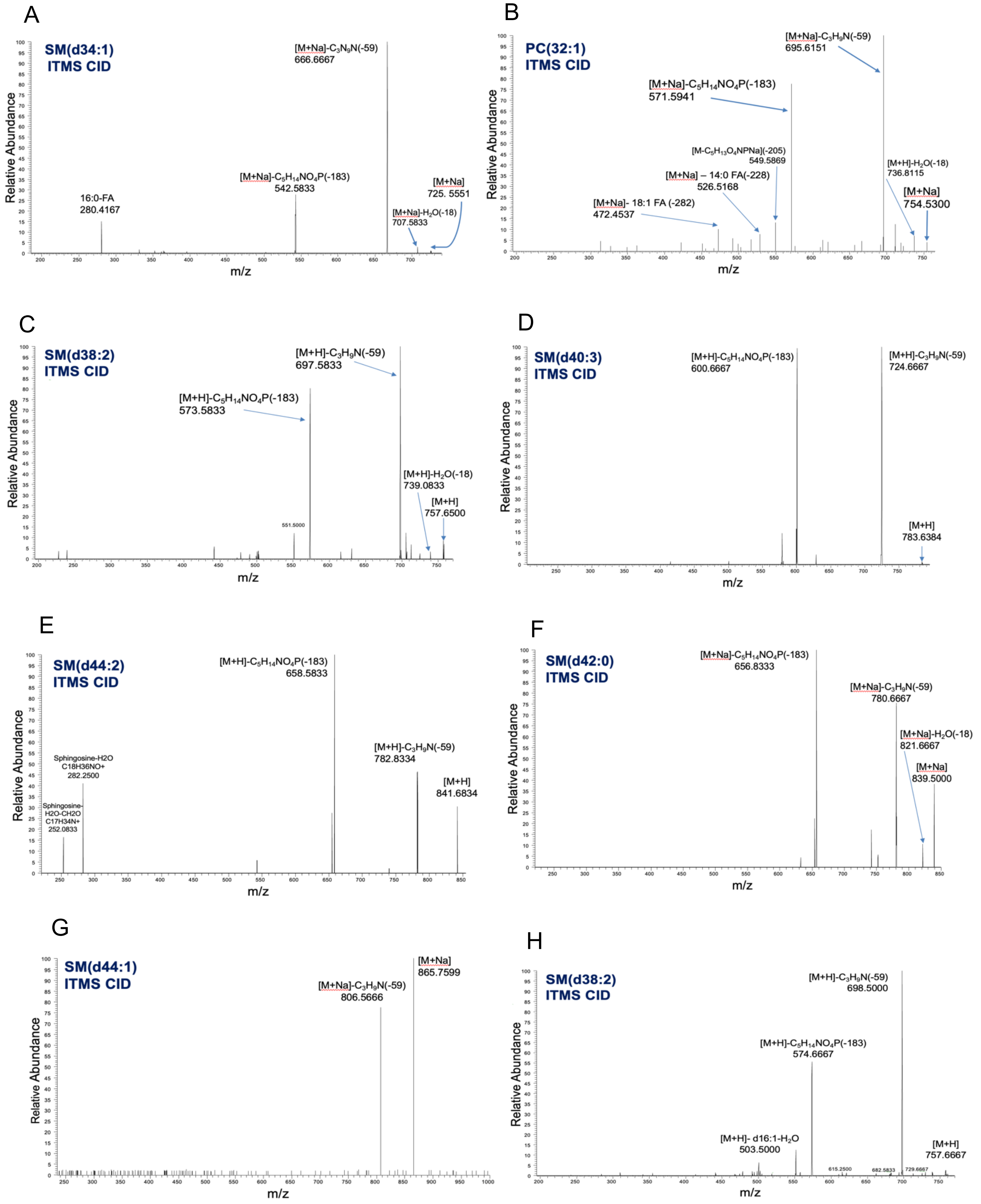

Fig. S10B. MS[2] identification of ions of interest from cell lysate (via HPLC-MS/MS) using ion- collision-induced dissociation (CID) with ion trap mass analyzer (ITMS) in the positive ion mode. (A) SM(d34:1), (B) PC(32:1), (C) SM(d38:2), (D) SM(d40:3), (E) SM(d44:1), (F) SM(d38:2).

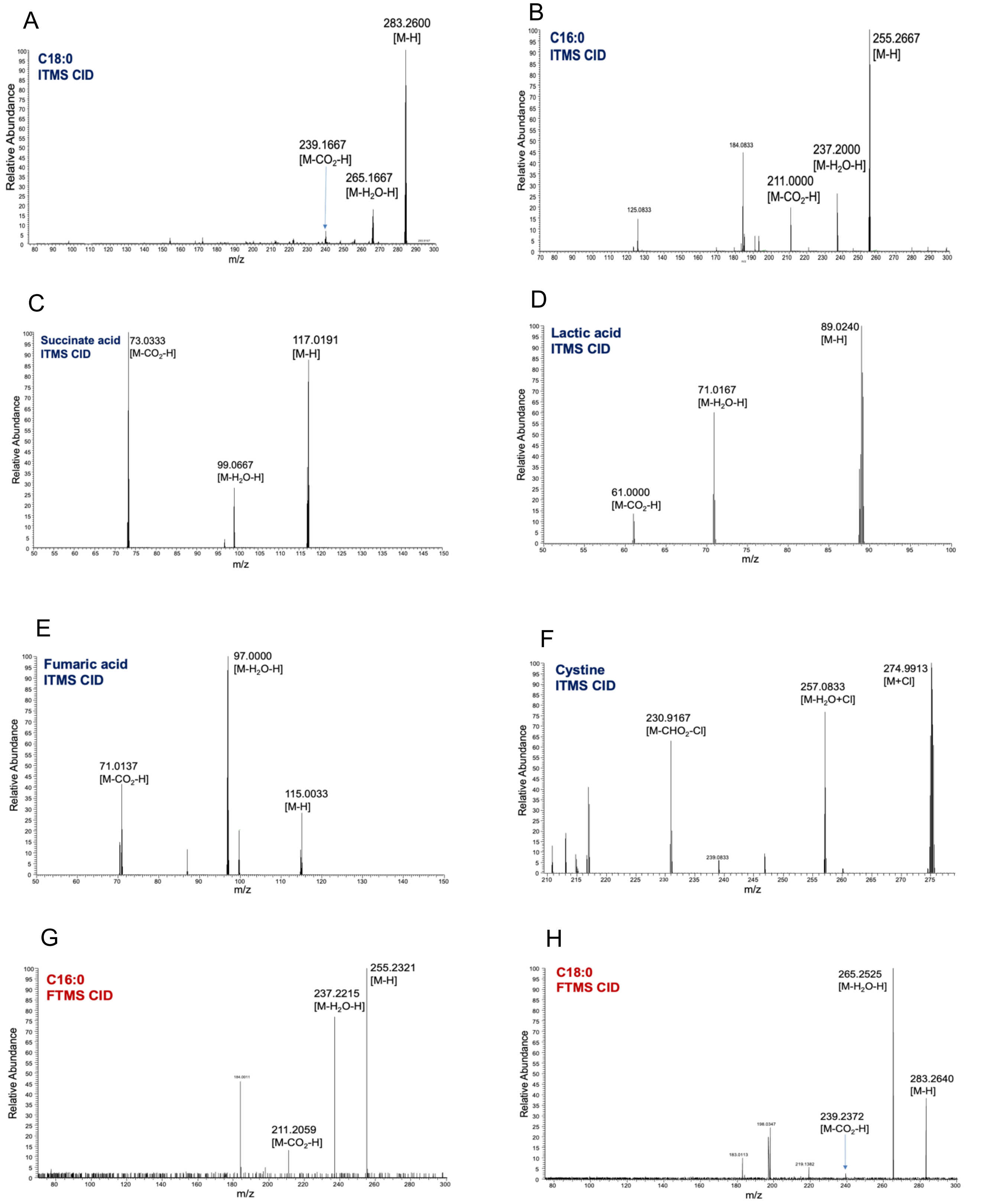

Fig. S11. MS^2 identification of ions of interest with CID from cell lysate in the negative ion mode. Data collection was performed using ion-trap (ITMS, A-F) or Orbitrap (FTMS, G-H) for (A) C18:0, (B) C16:0, (C) Succinate acid, (D) Lactic acid, (E) Fumaric acid, (F) Cystine. The collision-induced dissociation (CID) with orbitrap mass analyzer (FTMS) was used to confirm the structures of (G) C16:0 and (H) C18:0.
